# Household Food Allergen Exclusion Practices and Food Allergy-Related Psychosocial Functioning

**DOI:** 10.1001/jamanetworkopen.2024.52646

**Published:** 2024-12-27

**Authors:** Hana B. Ruran, Gabrielle D’Ambrosi, Roxanne Dupuis, Linda J. Herbert, Scott H. Sicherer, Wanda Phipatanakul, Lisa M. Bartnikas

**Affiliations:** 1 Case Western Reserve University School of Medicine, Cleveland, Ohio; 2 Division of Immunology, Department of Medicine, Boston Children’s Hospital, Harvard Medical School, Boston, Massachusetts; 3 Biostatistics and Research Design Center, Boston Children’s Hospital, Boston, Massachusetts; 4 Department of Population Health, New York University Grossman School of Medicine, New York; 5 Division of Psychology and Behavioral Health, Children’s National Hospital, Washington, DC; Department of Pediatrics, George Washington University School of Medicine, Washington, DC; 6 Division of Allergy and Immunology, Department of Pediatrics, Icahn School of Medicine at Mount Sinai, New York City, New York

## Abstract

This cross-sectional study examines households that exclude allergens by specific food allergy and the association of food allergy-related psychosocial functioning.

## Introduction

Food allergy (FA) affects 10% of children worldwide.^1,2^ FA causes stress and quality of life (QOL) concerns associated with accidental exposure and emergency treatment.^3,4^ Some parents manage their child’s FA by excluding allergens from their home. We sought to determine the proportion of households excluding allergens by specific FA and its association with this practice and FA-related psychosocial functioning.

## Methods

This cross-sectional study followed the American Association for Public Opinion Research (AAPOR) reporting guideline and was approved the Boston Children's Hospital institutional review board (IRB). IRB-approved validated surveys^5^ and informed consent were collected anonymously from families of children with health care clinician–diagnosed FA from Boston Children’s Hospital and social media outlets from April 2022 to November 2023. The number of potential participants reached is unknown.

We used logistic regression with generalized estimating equations to account for individual clustering to compare household FA exclusion practices by specific FA. Psychosocial comparisons were made between those with household food exclusions vs those without. Two-sided *P* < .05 were considered statistically significant (eMethods in Supplement 1). Analyses were conducted with SAS version 9.4 (SAS Institute).

## Results

A total of 919 surveys were completed by parents of a child with FA from 39 US states and Canada. The median (IQR) parent age was 39 (36.0-43.0) years and child age was 6 (3.0-9.0) years. Among parents, 881 were mothers (96.6%), 23 were Black individuals (2.5%), and 775 were White individuals (85.5%). Among children, 521 were males (57.6%), 29 were Black children (3.2%), and 704 were White children (78.6%). Most parents (586 [63.8%]) excluded at least 1 food from their home because of their child’s FA.

The most common allergies were peanut (623 [67.8%]), tree nut (602 [65.5%]), and egg (404 [44.0%]). Based on a priori analyses, the foods most excluded from homes with FAs were peanut (389 [62.4%]), tree nut (329 [54.7%]), and sesame (120 [51.3%]). Comparatively, only 98 homes with an egg allergy (24.3%) excluded egg from the home. The difference in the proportion of those excluding sesame compared with those excluding egg, milk, soy, and wheat were significant (Figure).

**Figure.**
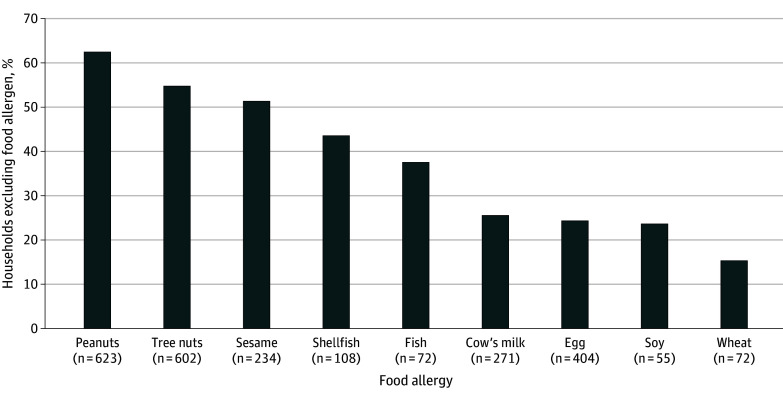
Proportion of Households That Excluded a Food Allergen of All Households With a Child With That Food Allergy

Parents who excluded food from the home because of their child’s FA reported worse mean (SD) FA-related QOL scores for worry (2.6 [1.5] vs 1.7 [1.4]), anxiety (34.3 [23.6] vs 16.9 [15.6]), and self-efficacy (79.7 [14.1] vs 84.8 [13.4]) compared with parents who did not (*P* < .001). These findings were consistent in age subgroups; parents who excluded their child's FA had higher mean (SD) worry scores than those who did not (child age 4 to 6 years: 2.1 [1.5] vs 1.7 [1.6]; *P* = .02; child age 7 to 12 years: 3.2 [1.6] vs 1.9 [1.3]; *P* < .001).The parent-proxy screener for child anxiety disorders indicated that children aged 8 to 17 years living in homes that exlcuded food allergens were more likely to have elevated generalized anxiety scores (42 of 138 children [30.4%] vs 10 of 64 children [15.6%]) than children from homes that did not exclude food-related allergens (*P* = .03) (Table).

**Table.** Parent and Child Psychosocial Well-Being by Household Allergen Exclusion Practice

Psychosocial well-being measure	One or more of child’s food allergen(s) excluded from home (n = 586)		Child’s food allergen(s) not excluded from home (n = 332)		*z *Score	*P* value
	Respondents, No. (%)	Mean (SD)	Respondents, No. (%)	Mean (SD)		
**Parent measures**						
FAQL-PB^a^^,^^b^	541 (92.3)	2.6 (1.5)	298 (89.8)	1.7 (1.4)	−8.83	<.001
FAIM-PF^a^^,^^b^	457 (78.0)	4.0 (0.9)	251 (75.6)	3.5 (1.0)	−6.59	<.001
WAFA2-P^c^						
Preschool	214 (36.5)	28.8 (22.3)	134 (40.4)	18.7 (17.1)	−4.19	<.001
Child	184 (31.4)	34.3 (23.6)	76 (22.9)	16.9 (15.6)	−5.72	<.001
Teen	56 (9.6)	30.7 (26.7)	23 (6.9)	29.3 (24.4)	−0.58	.56
FASE-P^a^^,^^d^	417 (71.2)	79.7 (14.1)	231 (69.6)	84.8 (13.4)	5.25	<.001
Elevated SCAARED, No. (%)^e^	511 (87.2)	193 (37.8)	274 (82.5)	85 (31.0)	3.55 (1)^f^	.06
**Child measures**						
FAQLQ^g^^,^^b^						
Parent form						
Child aged 0-3 years	349 (59.6)	3.6 (5.7)	193 (58.1)	4.8 (6.2)	1.8	.06
Child aged 4-6 years	129 (22.0)	2.1 (1.5)	62 (18.7)	1.7 (1.6)	−2.3	.02
Child aged 7-12 years	125 (21.3)	3.2 (1.6)	60 (18.1)	1.9 (1.3)	−6.01 (144)^h^	<.001
Child form, aged 8-12 years	37 (6.3)	4.7 (1.7)	15 (4.5)	3.6 (1.6)	−2.27	.02
Teen form, aged 13-17 years	26 (4.4)	4.9 (1.3)	10 (3.0)	4.4 (1.3)	−1.01 (34)^h^	.32
FAIM-CF^b^^,^^d^	60 (10.2)	3.9 (1.8)	23 (6.9)	3.5 (1.1)	−1.40 (81)^h^	.17
WAFA2^e^						
WAFA2-Child	33 (5.6)	26.1 (24.7)	14 (4.2)	14.0 (17.5)	−1.54	.12
WAFA2-Teen	25 (4.3)	20.3 (21.9)	9 (2.7)	21.6 (27.4)	0	>.99
Elevated SCARED^i^						
Parent proxy, ages 8-17 years	138 (23.5)	42 (30.4)	64 (19.3)	10 (15.6)	5.02 (1)^f^	.03
Child version, ages 8-17 years	62 (10.6)	15 (24.2)	23 (6.9)	5 (21.7)	0.06 (1)^f^	.81

Abbreviations: FAIM-CF, Food Allergy Independent Measure-Child Form; FAIM-PF, Food Allergy Independent Measure-Parent Form; FAQL-PB, Food Allergy Quality of Life-Parental Burden; FAQLQ, Food Allergy Quality of Life Questionnaire; FASE-P, Food Allergy Self-Efficacy Scale for Parents; SCAARED, Screen for Adult Anxiety Related Disorder; SCARED, Screen for Child Anxiety Related Disorders; WAFA2, Worry About Food Allergy 2.0; WAFA2-P; Worry About Food Allergy 2.0-Parent.

^a^ Only those who responded to every item or are missing only 1 item for this measure were included in the calculation.

^b^ A higher score indicates worse food allergy-related quality of life.

^c^ A higher score indicates greater food allergy-related anxiety.

^d^ A lower score indicates worse food allergy-related self-efficacy.

^e^ A score of 23 or greater may indicate the presence of an anxiety disorder (cutoff for binary variable).

^f^ *t* Test.

^g^ Only those who responded to every item for this measure were included in the calculation.

^h^ χ^2^ Test statistic, and degrees of freedom = 1.

^i^ A score of 25 or greater may indicate the presence of an anxiety disorder (cut-off for binary variable).

## Discussion

In this study, most families chose to exclude food allergens from their household and families engaging in this practice reported more FA-related psychosocial concerns than families who did not. Our findings suggest that insight about the psychosocial well-being of a family could be obtained if clinicians asked how FAs were managed in the home. Additionally, allocating time during appointments to discuss ways to manage FAs may help decrease stress and anxiety for children with FAs. Other studies^1,3,6^ have found that psychosocial distress may be experienced in greater proportions in families of children with peanut, tree nut, and sesame allergies compared with families of children with other FAs, such as egg and milk, which are equally important, more prevalent, and potentially life-threatening.

Our study is limited by its cross-sectional design and causality between psychosocial well-being and excluding allergens from the home cannot be determined and may not represent the broader FA population. Future studies are needed to determine the causes of psychosocial distress in families who exclude FA from the home and how the psychosocial well-being of families of children with FA can be improved.

## Notes

Supplement 1.
eMethods.

Supplement 2.
Data Sharing Statement
