# Supplementary material for: Household Food Allergen Exclusion Practices and Food Allergy-Related Psychosocial Functioning
Source: JAMA Netw Open. 2024 Dec 27;7(12):e2452646. doi: 10.1001/jamanetworkopen.2024.52646 (PMC11681370; doi:10.1001/jamanetworkopen.2024.52646)
Supplement: Supplement 1. — eMethods. [file jamanetwopen-e2452646-s001.pdf]

Supplemental Online Content

Ruran HB, D’Ambrosi G, Dupuis R, et al. Household food allergen exclusion practices and food allergy-related psychosocial functioning. *JAMA Netw Open*. 2024;7(12):e2452646. doi:10.1001/jamanetworkopen.2024.52646

eMethods

This supplemental material has been provided by the authors to give readers additional information about their work.

© 2024 Ruran HB et al. *JAMA Network Open.*

eMethods.

A logistic regression model with an identity link in the generalized estimating equations framework was estimated to account for individual clustering to compare household allergen exclusion differences by specific food allergy. For each comparison, the outcome was modeled as whether the respective allergen was excluded from the household and the predictor was the food allergy. Psychosocial comparisons were made between those with household food exclusions versus those without. Two-sided P values <0.05 were considered statistically significant.

© 2024 Ruran HB et al. *JAMA Network Open.*
