# Supplementary material for: Household Food Allergen Exclusion Practices and Food Allergy-Related Psychosocial Functioning
Source: JAMA Netw Open. 2024 Dec 27;7(12):e2452646. doi: 10.1001/jamanetworkopen.2024.52646 (PMC11681370; doi:10.1001/jamanetworkopen.2024.52646)
Supplement: Supplement 2. — Data Sharing Statement [file jamanetwopen-e2452646-s002.pdf]

**Data Sharing Statement**

**Data**
**Data available:** Yes
**Data types:** Deidentified participant data
**How to access data:** Requested data may be shared with researchers/collaborators whose proposed use of the data has been approved
**When available:** With publication

**Supporting Documents**
**Document types:** None

**Additional Information**
**Who can access the data:** Researchers whose proposed use of the data has been approved
**Types of analyses:** Proposed analyses for use of the data that has been approved
**Mechanisms of data availability:** Without investigator support, after approval of a proposal, and with signed data access agreement
